# Pancreatic Injury in Patients with SARS-Cov-2 (COVID-19) Infection: A Retrospective Analysis of CT Findings

**DOI:** 10.1155/2021/5390337

**Published:** 2021-10-31

**Authors:** Gabriela Grusova, Radan Bruha, Bianka Bircakova, Matej Novak, Lukas Lambert, Pavel Michalek, Grus Tomas, Andrea Burgetova

**Affiliations:** ^1^4th Department of Internal Medicine, First Faculty of Medicine, Charles University and General University Hospital in Prague, Prague, Czech Republic; ^2^Department of Radiology, First Faculty of Medicine, Charles University and General University Hospital in Prague, Prague, Czech Republic; ^3^Department of Anesthesiology and Intensive Care, First Faculty of Medicine, Charles University and General University Hospital in Prague, Prague, Czech Republic; ^4^2nd Department of Surgery-Department of Cardiovascular Surgery, First Faculty of Medicine, Charles University and General University Hospital in Prague, Prague, Czech Republic

## Abstract

**Objective:**

To determine the association between COVID-19 infection and peripancreatic changes on CT as a sign of acute pancreatic injury.

**Methods:**

Retrospective analysis of CT examinations in patients with confirmed COVID-19 infection yielded 103 instances. An age- and gender-matched cohort of patients without COVID-19 was found. CT examinations were evaluated for peripancreatic stranding or edema, fluid collection, or necrosis, without any other explanation. Depicted pulmonary parenchyma was evaluated for possible COVID-19-related changes. Clinical and laboratory data were retrieved from the clinical database.

**Results:**

Peripancreatic fat stranding (*n* = 8) or fluid collection (*n* = 2) without any other cause was found in 10 (10%) patients. Abdominal complaints were reported in 4 (40%) patients. Elevated serum amylase or lipase levels were documented in 5 (50%) patients who also satisfied the diagnostic criteria for acute pancreatitis. From the study sample of 103 patients with COVID-19, pulmonary parenchyma was depicted in 102 (99%), and from these, 57 (55%) had an evidence of pulmonary changes compatible with COVID-19 pneumonia. This proportion was not significantly different between patients with and without peripancreatic changes (*p* = 0.35). In the matched cohort, we found peripancreatic changes in 2 (2%, *p* = 0.033) patients. Patients with pancreatic injury and elevated amylase levels were more likely to require orotracheal intubation (35% vs. 12%, *p* = 0.021).

**Conclusions:**

We showed that the prevalence of peripancreatic stranding or fluid collection is higher in patients diagnosed with COVID-19 infection compared to an age- and gender-matched cohort. Patients with pancreatic injury and elevated amylase levels are more likely to require orotracheal intubation. Our findings corroborate the link between COVID-19 infection and pancreatic injury from the perspective of imaging.

## 1. Introduction

Data from the 2020 outbreak of SARS-CoV-19 (COVID-19) showed the association between gastrointestinal symptoms and COVID-19 infection in up to 79% of the patients [1]. These include nausea, vomiting, diarrhea, and abdominal pain [1, 2]. Wang et al. from the University Hospital in Wuhan, China, identified 9 (17%) patients with pancreatic injury defined as any abnormality in amylase or lipase levels [3].

Although this association has been confirmed by other authors [4–6], the link between COVID-19 infection and acute pancreatitis is not solid [7, 8]. In most patients, the levels of pancreatic enzymes are not elevated substantially, and these patients only occasionally develop symptoms to satisfy the diagnostic criteria of acute pancreatitis. There are scattered reports of COVID-19-related acute pancreatitis [9–11]. We were prompted to investigate this association by an unusual finding of peripancreatic edema without a known cause in three patients with COVID-19 at a single department at one time.

The aim of this study was to determine the association between COVID-19 infection and peripancreatic fat edema, stranding, or fluid collection as a sign of acute pancreatic injury.

## 2. Methods

This project was approved by the Ethics Committee of the General University Hospital in Prague (ref. 2221/20 S-IV); it was performed in accordance with the Declaration of Helsinki. Informed consent was waived due to the retrospective nature of the study.

**Figure 1:**
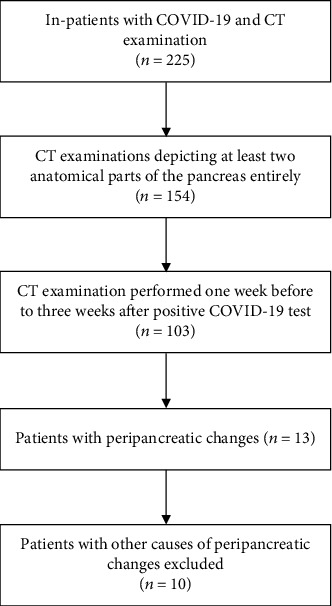
Study flow diagram.

We searched the hospital database for patients who were admitted to our tertiary care hospital between April 01 and November 30, 2020, who had a confirmed diagnosis of COVID-19 by real-time reverse-transcriptase polymerase chain reaction (rRT-PCR) assay and underwent CT examination during the hospital stay. The search yielded 225 patients from whom 154 had at least two anatomical parts of the pancreas depicted (Figure 1). From these, there were 103 patients with CT examination performed no earlier than one week and no later than three weeks from the first positive COVID-19 test [12]. For these patients, we found a 1 : 1 age- and gender-matched cohort of 103 patients without a diagnosis of COVID-19, who underwent CT examination during the same period.

Patients with other conditions that would induce peripancreatic edema (intraabdominal infection of another origin, postoperative conditions, hydronephrosis, and anasarca) were not considered.

In both cohorts, CT images were evaluated for peripancreatic fat edema and stranding (“stranding”), or (peri)pancreatic fluid collection and/or necrosis (“collection”) by two radiologists (one junior and one senior) in consensus. In patients with COVID-19, the clinical database was searched for selected items in medical history, laboratory data (amylase, lipase), and outcome.

The statistical analysis was performed in Prism v. 5.0. (GraphPad Software, La Jolla, CA). Categorical values were compared using the *F* test, continuous variables by *t*-test, or Mann–Whitney test according to their distribution (D'Agostino-Person normality test). A *p* value below 0.05 was considered significant.

## 3. Results

From patients with COVID-19, 58 had CT examinations, where the pancreas was depicted entirely. In 45 patients, at least two anatomical parts of the pancreas (the head, body, and tail) were depicted entirely. Of these 103 patients, 71 (69%) were males. Their age was 66 ± 14 years (Table 1).

**Figure 2:**
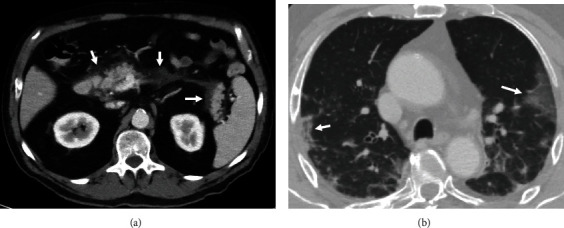
Acute peripancreatic fluid collection (a) in a patient with COVID-19 infection and pulmonary involvement (b).

Peripancreatic fat stranding (*n* = 8) or fluid collection (*n* = 2, Figure 2) without any other cause was found in 10 (10%) patients (age, 59 ± 16 years; 8 males). These patients had all three parts of the pancreas depicted. Abdominal complaints were reported in 4 (40%) patients. Elevated serum amylase or lipase levels were documented in 5 (50%) patients who also satisfied the diagnostic criteria for acute pancreatitis. None of the patients had gallstones, a history of chronic pancreatitis, alcohol or food excess, or hypertriglyceridemia.

From the study sample of 103 patients with COVID-19, pulmonary parenchyma (at least basal parts) was depicted in 102 (99%), and from these, 57 (55%) had evidence of pulmonary changes compatible with COVID-19 pneumonia. This proportion was not different between patients with (7 of 10; 70%). and without peripancreatic changes (50 of 93; 54%, *p* = 0.33).

The clinical characteristics of patients with peripancreatic changes did not significantly differ from those without—notably the body mass index (25.2 ± 3.4 vs. 27.6 ± 4.4 kg‧m^−2^, *p* = 0.92) and diabetes (3 of 10 (30%) vs. 24 of 93 (26%), *p* = 0.70). A more frequent requirement for orotracheal intubation (4 of 10 (40%) vs. 13 of 93 (14%)) did not reach statistical significance by a small margin (*p* = 0.058). When the group of patients with pancreatic injury was extended to those with elevated amylase levels, the difference (7 of 20 (35%) vs. 10 of 83 (12%)) became significant at *p* = 0.021. No difference between the groups was observed in the duration of hospital stay (17 (range 5–55) vs. 14 (range 1–75) days, *p* = 0.70) or the inhospital mortality (2 of 10 (20%) vs. 15 of 93 (16%), *p* = 1.0).

Demographic and clinical data from the hospital stay are presented in Tables 1 and 2.

In the matched cohort (age 67 ± 15 years), we found peripancreatic changes in 2 (2%, *p* = 0.033) patients aged 64 and 36 years (a male and a female). Neither of them had amylase or lipase levels sampled and neither reported abdominal pain.

## 4. Discussion

This study showed that the prevalence of peripancreatic stranding or fluid collection was higher in patients diagnosed with COVID-19 infection compared to an age- and gender-matched cohort. Elevation of pancreatic enzymes was linked with a more frequent need for orotracheal intubation.

Pancreatic injury from COVID19 has been explained by the expression of angiotensin-converting enzyme 2 (a target receptor for COVID19) in pancreatic islet cells [13]. Apart from the direct cytopathogenic effect of COVID-19, a systemic inflammatory response, drug-related pancreatic injury, or secondary immune-mediated inflammatory response has been suggested in its pathogenesis [3, 9, 14]. Whilst the link between COVID-19, hypercoagulability, and venous thromboembolism has been firmly established, splanchnic thrombosis has not been indicated in the pathogenesis of pancreatic injury [15].

In their series of 52 patients with COVID-19 from the University Hospital in Wuhan, China, Wang et al. identified 9 (17%) patients with (clinically inapparent) pancreatic injury defined as any abnormality in amylase or lipase levels [3]. Barlass et al. found markedly elevated lipase levels in 14 of 83 (17%) patients who tested positive for COVID-19 and were admitted to the hospital [4]. The association between elevated lipase levels as a marker of pancreatic injury and COVID-19 infection in up to 20% of patients has been reported by others as well [4–6, 16]. Inamdar et al. showed that in patients with COVID-19, the etiology of pancreatitis is mostly unknown (69% patients) in contrast to non-COVID-19 patients, where alcohol consumption and gallstones are the main culprits [17].

Our study, which approached the association between COVID-19 and pancreatic injury from the perspective of clinical imaging, showed that 9.7% of COVID-19 patients had evidence of pancreatic injury of unknown cause on CT. This is in line with the observation of Liu et al., who reported that 5 of 67 (7.5%) patients treated for severe COVID-19 in Wuhan, China, had detectable changes on CT indicating pancreatic inflammation (pancreatic enlargement and dilation of the pancreatic duct, but not pancreatic necrosis) [13]. The proportion of patients with peripancreatic changes in our and Liu's study was lower because elevated pancreatic enzyme levels are more sensitive to pancreatic injury than CT imaging. They are also more sensitive than the presence of typical clinical symptoms. Moreover, patients with COVID-19-related pancreatic injury often report nonspecific gastrointestinal symptoms (nausea, anorexia, diarrhea, and abdominal discomfort but not pain) [18]. In debilitated and unconscious patients, these symptoms may not be recognized, which may result in underreporting the prevalence of COVID-19-related pancreatic injury.

Patients with COVID-19 infection may exhibit elevation of serum levels of pancreatic enzymes, but they may not develop clinical symptoms or otherwise satisfy the diagnostic criteria for acute pancreatitis [19]. This is also the reason why cases of COVID-19-induced pancreatitis are scattered [10]. The clinical significance of increased lipase levels in patients with COVID19 has been questioned [8, 20]. There is still a lack of evidence to confirm the association between COVID-19 and acute pancreatitis [7]. A case-control study by Miró et al. reported a lower frequency of acute pancreatitis of any etiology in patients with COVID19 attending the emergency department compared to non-COVID19 patients [21].

The clinical characteristics of patients with peripancreatic changes did not significantly differ from those without. Even a more frequent requirement for orotracheal intubation did not reach statistical significance by a small margin. The increased need for mechanical ventilation in patients with pancreatic injury (based on ultrasound and serum enzyme levels) has been reported by Ding et al. with an odds ratio of 10.2 [22]. When we extended the group of patients with pancreatic injury to those with elevated amylase levels, the difference became significant too. Naturally, in Ding's study, pancreatic injury was also associated with increased mortality and the duration of the hospital stay, which we could not confirm.

### 4.1. Study Limitations

This is a retrospective study that suffers from the selection bias because only patients with confirmed COVID-19 infection who required hospital admission and CT imaging for any indication that covered the pancreas were included. We assume that the prevalence of peripancreatic changes in an unselected population would be lower. Secondly, the serum levels of pancreatic enzymes were not available in about half of the patients because the pancreatic injury was not suspected. Thirdly, the comparison between patients with and without peripancreatic changes is underpowered due to a small number of subjects with peripancreatic changes. Analysis of clinical, imaging, and laboratory from large multicenter databases will be required to corroborate the link between COVID-19 infection and pancreatic injury and its clinical significance.

In conclusion, we showed that the prevalence of peripancreatic stranding or fluid collection is higher in patients diagnosed with COVID-19 infection compared to an age- and gender-matched cohort. Our findings corroborate the link between COVID-19 infection and pancreatic injury from the imaging perspective.

## Figures and Tables

**Table 1:** Demographic data of patients.

	Patients with COVID-19 and peripancreatic changes (*n* = 10)	Patients with COVID-19 and normal pancreas (*n* = 93)	*p*	Patients without COVID-19 and with peripancreatic changes (*n* = 2)
Gender (male)	8 (80%)	63 (68%)	0.72	1 of 2
Age (years)	59 ± 16	66 ± 13	0.095	36, 65
BMI (kg·m^−2^)	25.2 ± 3.4	27.6 ± 4.4	0.092	24.5, 30.8
Comorbidities (*n*)				
Diabetes	3 (30%)	24 (26%)	0.70	0
Renal failure	3 (30%)	13 (14%)	0.15	1
Hypertension	5 (50%)	59 (63%)	1.0	1
Ischemic heart disease	1 (10%)	19 (20%)	0.68	1
Chronic pulmonary disease	1 (10%)	17 (18%)	1.0	0

**Table 2:** Clinical data from the hospital stay.

	Patients with COVID-19 and peripancreatic changes (*n* = 10)	Patients with COVID-19 and normal pancreas (*n* = 93)	*p*	Patients without COVID-19 and with peripancreatic changes (*n* = 2)
Duration of hospital stay (days)	17 (5–55), median (range)	14 (1–75), median (range)	0.70	42, 2
Elevated amylase or lipase (*n*)	6 (60%)	10 (11%)	0.0008	0
CT findings (*n*)				
Peripancreatic stranding	8 (80%)	0	<0.0001	2
Fluid collection	2 (20%)	0	0.0086	0
Ventilatory support (*n*)				
None or oxygen	6 (60%)	78 (84%)	0.084	1
NIV	0	2 (2%)	1.0	0
OTI	4 (40%)	13 (14%)	0.058	1
Thromboembolic events (*n*)				
Pulmonary embolism	2 (20%)	4 (4%)	0.10	0
Deep vein thrombosis	0	4 (4%)	1.0	0
Stroke	0	2 (1%)	1.0	0
Outcome (*n*)				
Died	2 (20%)	15 (16%)	1.0	1
Discharged or transferred to a local hospital	9 (90%)	78 (84%)	1.0	1

NIV: noninvasive ventilation; OTI: orotracheal intubation.

## Data Availability

Data is available on request.
